# Assessing the Contribution of Managerial Accounting in Sustainable Organizational Development in the Healthcare Industry

**DOI:** 10.3390/ijerph20042895

**Published:** 2023-02-07

**Authors:** Anca Antoaneta Vărzaru, Claudiu George Bocean, Maria Magdalena Criveanu, Adrian-Florin Budică-Iacob, Daniela Victoria Popescu

**Affiliations:** 1 Department of Economics, Accounting and International Business, University of Craiova, 200585 Craiova, Romania; 2 Department of Management, Marketing and Business Administration, University of Craiova, 200585 Craiova, Romania

**Keywords:** managerial accounting, accounting roles, sustainability, digital transformation, digital technologies

## Abstract

Sustainability and digital transformation are two phenomena influencing the activities of all organizations. Managerial accounting is an essential component of these transformations, having complex roles in decision-making to ensure sustainable development through implementing modern technologies in the accounting process. This paper studies the roles of digitized managerial accounting in organizational sustainability drivers from a decision-making perspective. The empirical investigation assesses the influence of managerial accounting on the economic, social, and environmental drivers of sustainability from the perception of 396 Romanian accountants using an artificial neural network analysis and structural equation modeling. As a result, the research provides a holistic view of the managerial accounting roles enhanced by digital technologies in the sustainable development of healthcare organizations. From the accountants’ perception, the leading managerial accounting roles on organizational sustainability are enablers and reporters of the sustainable value created in the organization. Additionally, the roles of creators and preservers are seen as relevant by a significant part of the respondents. Therefore, healthcare organizations must implement a sustainability vision in managerial accounting and accounting information systems using the capabilities offered by new digital technologies.

## 1. Introduction

Organizations must improve accounting information systems to be sustainable using new digital technologies, such as cloud computing, big data, artificial intelligence, and the internet of things. This way, organizations can correctly inform and report objectively on strategies, objectives, and organizational activities. In addition, a sustainable orientation based on digital transformation will improve communication with key stakeholders, attract customer loyalty, and improve image and reputation, constituting a real competitive advantage [1,2,3].

Organizational reporting influences financial stability and sustainable development, with accountants essential in this process. Managerial accounting evaluates and discloses all the necessary information concerning the sustainability of organizational activities. The role of managerial accounting professionals in sustainable value management is increasing [3]. Managerial accounting professionals are increasingly active in integrating sustainability into organizational strategy and practice, and digital technologies can facilitate this role. The academic literature offers many exploratory studies in this field, but empirical studies are few, which constitutes a research gap. Therefore, perception studies among managerial accounting professionals are necessary [4] to better assume managerial accounting roles in ensuring organizational sustainability. This paper aims to cover this gap through a study carried out within the healthcare industry.

This paper aims to define the roles of digitized managerial accounting in decision-making to ensure the organization’s sustainable development and evaluate the most important roles in the perception of accountants. Schaltegger and Zvezdov “suggests that the accountant’s role in social and environmental accounting needs to be explored in corporate practice” [5] (p. 334). The empirical study of the researched problem was carried out in healthcare organizations in Romania. The two research questions emerging from the research purpose are RQ1. What are the most significant roles of digitized managerial accounting in the sustainable value creation process perspective? RQ2. What is the influence of managerial accounting on sustainable development and the mediating effect of the digital transformation of managerial accounting? The empirical investigation answering these questions uses an artificial neural network analysis and structural equation modeling, providing a holistic view of the managerial accounting roles enhanced by digital technologies on the sustainable development of healthcare organizations.

## 2. Literature Review

The global economic environment, which has become increasingly volatile in recent years, has affected the ability of organizations to adapt to the conditions to survive in the context of a growing demand for sustainability [1,6,7,8]. Managerial accounting can be the answer through which the accounting and reporting of the sustainability of organizations can be organized effectively, using new digital technologies that have generated a digital transformation of business models. Furthermore, integrating managerial accounting tools in organizational performance management systems leads to better management and control of the organization’s sustainable performance [9].

A company’s ability to generate qualitative information about the activities carried out in terms of sustainability determines the management decision efficiency [10]. Although research concerning managerial accounting roles in sustainability has been a subject of study in recent decades, the empirical evidence has been mainly based on case studies in developed countries [11,12].

Nevertheless, the academic literature has discussed the managerial accounting roles in ensuring sustainability. Various authors [13,14,15,16] have emphasized the need for a critical approach to understanding managerial accounting roles in sustainability and digital transformation. For example, Lovell and MacKenzie [17] analyzed the role of accounting professional organizations in managing the new economy characterized by climate change and digital transformation. Ngwakwe [18] critically examined sustainability initiatives in the accounting profession and demonstrated the need for a more pragmatic approach based on new digital technologies. Other researchers [5,19,20,21,22,23] have studied how managerial accounting can support organizational sustainability. Likewise, many researchers have also pointed out the role of managerial accounting in improving the sustainability performance by supporting managerial decisions in managing organizational changes [1,24,25,26,27].

The International Federation of Accountants (IFAC) [28,29] ranks the contribution of managerial accounting to the process of ensuring organizational sustainability through three decision-making levels of the organization: the strategic level, the operational level, and the reporting level. The strategic level involves the participation of managerial accounting in making strategic decisions and objective sets. The operational level implies a total involvement of managerial accounting in the budgeting process, cost calculations, and performance measurement activities, providing the financial, environmental, and social information necessary to ensure organizational sustainability. Finally, the reporting level involves the preparation of financial, sustainability, or integrated reports that provide a complete picture of an organization’s sustainable performance. The integration of sustainability vectors (financial, social, and environmental) at all levels of the decision-making process (strategic, tactical, and operational) and in all managerial functions (planning–budgeting, organization coordination, staffing, and evaluation) to improve stakeholder relations are crucial tasks for managerial accounting. Therefore, managerial accounting must participate more actively in creating and maintaining the sustainable value of the organization.

The sustainable value is a concept interpreted in various ways. In our paper, we defined sustainable value as identifying and managing a balance between ecological, social, and economic values [30], starting from the Triple Bottom Line approach that defines these three dimensions of sustainability [31,32]. Organizations must behave sustainably, pursuing social and environmental objectives while increasing shareholder values.

Managerial accounting supports organizations in meeting all their objectives, including sustainability. Schaltegger and Zvezdov [5] (p. 342) showed that “the accountants could … expected to play an important role, first, in improving the information stakeholders receive about the social and environmental impacts of a company and, second, in improving how well managers are informed about sustainability issues”. According to the IFAC [28,29], managerial accounting accountants fulfill the roles of creators, enablers, preservers, and reporters of sustainable value for their organizations. The role of sustainable value creators involves setting strategies, policies, and plans necessary to create a sustainable value. The role of enablers of the sustainable value presumes to inform and guide operational management decisions by improving support processes. The role of sustainable value preservers involves protecting sustainable value creation strategies by contributing to the risk management process. As reporters of sustainable value, accountants must ensure the transparent and fair communication of delivering a sustainable value. The accounting professional plays a crucial role in ensuring organizational performance and compliance with laws and good practices in the field of sustainability and in optimizing the balance between the two dimensions: profit maximization and ethical business behavior. Ethical considerations are even more important in the healthcare industry, with care for patients considered the most critical organizational goal [33,34,35,36,37,38,39,40,41,42].

The first research hypothesis based on the identified gap and the first research question (RQ1) is the following:

**Hypothesis H1.**  *In the perception of managerial accounting professionals, the roles of enablers and reporters are the most important in the sustainable value creation process perspective*.

Although many authors emphasize the role of managerial accounting in sustainability accounting and reporting, there is uncertainty about the parts of accountants in ensuring sustainability [43,44,45], suggesting the acquisition of new skill sets specific to sustainability recording, interpretation, and organizational reporting but also the use of new digital technologies. For example, Schaltegger [46] showed that managerial accounting professionals can solve sustainability issues more efficiently by integrating digitized measurement and management tools.

Willekes et al. [3] showed that it is increasingly necessary to integrate managerial accounting into sustainability accounting to create an efficient accounting information system that provides data not only on financial values but also on social and environmental values [47,48,49]. Furthermore, sustainability accounting and reporting must use digital transformation’s managerial accounting tools and innovations [5,22,23,50,51].

Many authors [52,53,54] have highlighted the importance of innovations of any type (including the implementation of digital technologies) in managerial accounting to contribute more to ensuring organizational sustainability. Other authors [55,56,57,58] have focused on the competencies needed by accountants in the context of digital transformation and the need to ensure organizational sustainability.

Digital technologies occupy an essential place in increasing the role of managerial accounting in an organizational strategy and, in particular, in ensuring organizational sustainability [59,60,61,62,63,64,65,66,67,68,69,70,71,72,73,74,75,76,77,78]. Cloud computing enables real-time information sharing and greater organizational transparency. The data collected directly using the sensors integrated into the internet of things technology are transferred to big data technologies and processed using IT solutions that incorporate artificial intelligence.

The second research hypothesis based on this assumption and the second research question (RQ2) is the following:

**Hypothesis H2.**  *Managerial accounting significantly influences sustainable development, with digital transformation exerting a strong mediating effect*.

## 3. Materials and Methods

**Figure 1:**
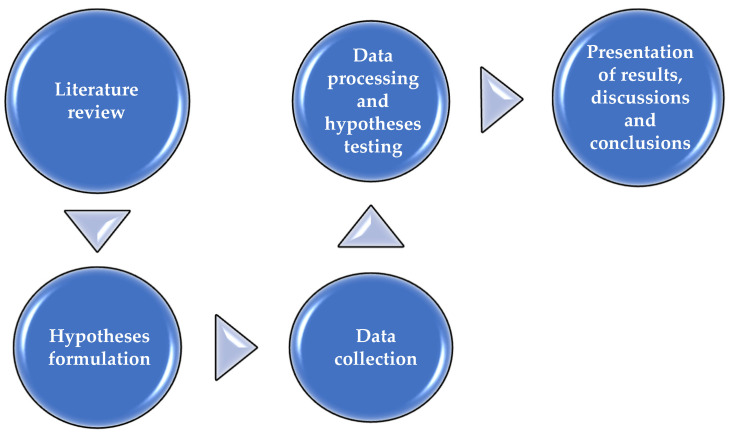
Research steps. Source: the authors’ design.

The research process took place in three steps, starting from the analysis of the academic literature to the presentation of the results and conclusions (Figure 1).

We used a questionnaire presented in Appendix A to analyze the perceptions of managerial accounting professionals in Romania concerning the managerial accounting roles in ensuring sustainability and the mediating effect exerted by digital transformation. The questionnaire includes general questions concerning the perceptions of professionals in managerial accounting and does not include data requiring institutional review boards. In the questionnaire, we used the Likert scale with five levels (Table 1).

The exogenous variables in the questionnaire (items) were selected based on empirical studies that used similar variables [2,5,15,18,28,29,32,45,51,59,65,66]. The main variables of the research were managerial accounting roles in creating the economic value of an organization proposed by the IFAC: “creators, enablers, preservers, and reporters” of value [28] (p. 15). For sustainable development, we used the three pillars (social, economic, and environmental) theorized by Elkington to synthesize the very complex concept of sustainability. According to the IFAC [79] (p 6.), “Sustainable value creation involves considering economic, environmental, and social factors—not only because different stakeholders have different interests, but also because these factors are interdependent. Environmental and social factors can also determine or affect the economic value of an organization”. Managerial accountants from health organizations were questioned on their roles in value creation to assess the influence on sustainable economic, environmental, and social success. The framework proposed by the IFAC was briefly presented in the preamble of the questionnaire. For digitization, we used a single synthesizing variable that concerns the degree of digital technologies used in the activity of managerial accountants in health organizations. The paper aims to determine the mediating effect of digital transformations in the relationship between managerial accounting roles and the sustainable development pillars. The survey was carried out between October and November 2022 in Romania. The sample is represented by 396 professionals in managerial accounting from the Southwest Oltenia Region healthcare organizations in Romania.

The study used the stratified random sampling method. In Romania, according to the National Institute of Statistics [80], in 2021, there were 535 healthcare organizations, of which 367 were in the public sector and 168 were in the private sector. The Southwest Oltenia Region is representative of the national level, being a region with an average level of development among the eight regions of Romania, which ensures representativeness from the point of view of economic development. In the Southwest Oltenia Region, in 2021, there were 10% of the total healthcare organizations (36 in the public sector and 17 in the private sector), which ensures representativeness from the point of view of ownership. The sample, selected from professionals in the managerial accounting of healthcare organizations, has a confidence level of 95%, with a margin of error of 4.67%. The layers setting depended on two demographic criteria (gender and age). Among the total respondents, 53.5% were male, and 46.5% were female. Furthermore, the structure depended on age: 26.3% of respondents were in the 18–30 years category, 43.4% were in the 31–45 years category, and 30.3% were between 46 and 65 years. Table 2 exposes the descriptive statistics.

**Figure 2:**
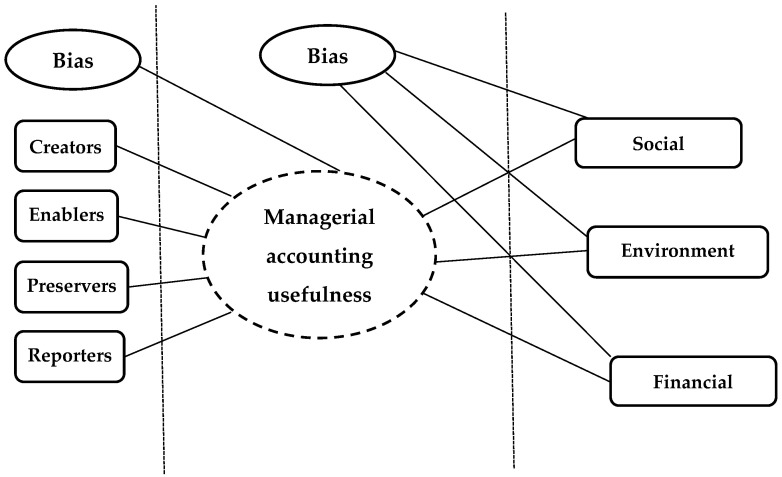
Conceptual model. Source: the authors’ design based on [2,15,28,29,32].

We used the artificial neural network analysis to test the first hypothesis, which allows for setting the influences between variables in two layers (an input layer and an output layer) [81,82]. The hidden layer between the two layers represents managerial accounting’s usefulness in organizational sustainability from the perception of managerial accounting professionals. Figure 2 illustrates the research model.

**Figure 3:**
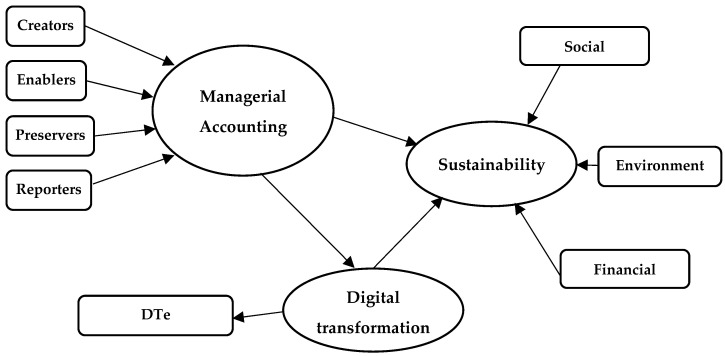
Theoretical model. Source: the authors’ design based on [2,15,28,29,32].

The testing of the second hypothesis of the research involved the use of structural equation modeling, which allows the assessment of the relationships established among the latent (endogenous) variables, variables determined based on the exogenous variables (questionnaire items) [83,84,85]. Figure 3 shows the research model.

## 4. Results

**Figure 4:**
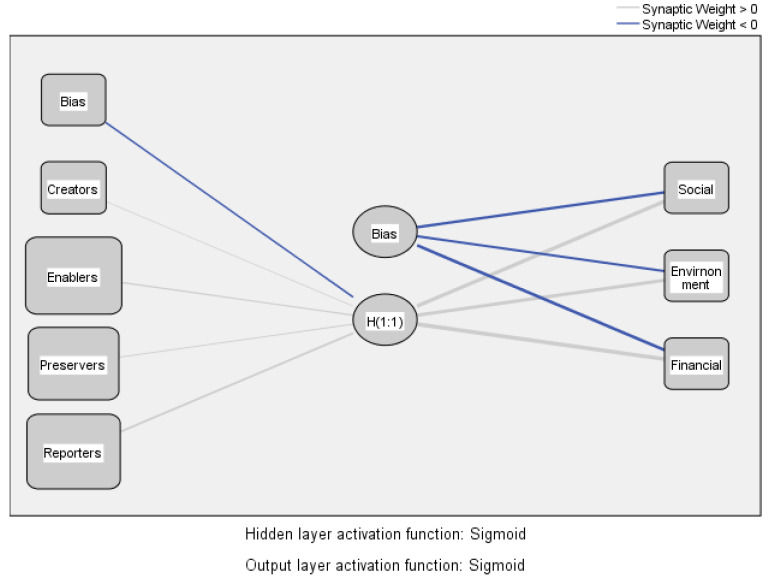
MLP model. Source: the authors’ design using SPSS v.20.

The testing of the H1 hypothesis involved the use of a multilayer perceptron (MLP) model in the artificial neural network (ANN) analysis. This model illustrates the influences between managerial accounting roles placed in the input layer and sustainability drivers placed in the output layer [81,82]. The activation functions in MLP are sigmoid types. The overall average relative error is 0.290. Figure 4 shows the relationships among the model variables.

Table 3 illustrates the MLP predictors and the variables’ importance.

Figure 4 and Table 3 show the significant influences of managerial accounting roles on the three drivers of sustainability. The financial driver remains the most critical driver of sustainability influenced by managerial accounting from the perception of managerial accountants in the healthcare industry. However, the model highlights significant influences on the social and environmental drivers. Among the roles of managerial accounting professionals in the healthcare industry, the most important are reporters, followed by enablers. The preservers and the creator roles significantly influence the three drivers of sustainability. Therefore, the results of the artificial neural analysis confirmed the validity of hypothesis H1. From managerial accounting professionals’ perceptions, enablers’ and reporters’ roles are the most important in the healthcare industry’s sustainable value creation process. The reporter role is traditional for accounting, but the enabler role shows that managerial accounting professionals are aware of its importance in decision-making to ensure sustainability by participating in sustainable value creation.

To investigate the influences of managerial accounting roles on the three pillars of sustainable development, we used a multivariate analysis of variance. Table 4 exposes the multivariate tests in MANOVA.

The Wilks’ Lambda test showed that the model was statistically significant. For all four managerial accounting roles, *p* values < 0.001. The observed power was 1.000 for all variables, highlighting a 100% chance for result significance. The size effect (partial eta squared values) indicated that enablers’ and reporters’ roles were the most important from the healthcare industry’s sustainable value creation process perspective.

To identify the influence of each role of managerial accounting on each pillar of sustainable development, we ran tests of between-subject effects (Table 5).

The analysis of the effects between the variables reveals that the roles of enablers and reporters are the ones that influence the social and economic pillars the most. The environment pillar is most influenced by the creator and reporter roles. The analysis shows that the traditional role of the reporter remains significant, but the influence of the enabler and creator roles is increasing. Digital transformation has an essential contribution to rethinking the roles of managerial accounting from the perspective of sustainable development.

**Figure 5:**
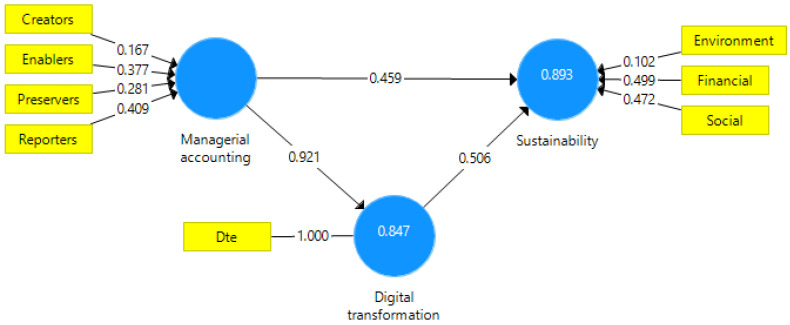
Applied model. Source: the authors’ own design using SmartPLS v3.0.

The mediating effect of digital transformation is analyzed using SEM-PLS (structural equation modeling–partial least squares). The software used for structural equation modeling (SmartPLS v3.0) to test hypothesis H2 allows using the PLS algorithm applied within a formative model with a small number of variables [83,84,85]. According to Hair et al. [85] (p. 11), “PLS-SEM can easily handle reflective and formative measurement models, as well as single-item constructs, with no identification problems”. The leading roles in the perception of managerial accounting professionals are enablers and reporters of sustainable values (Figure 5).

To test the reliability of the collected data series, we used Guttman’s reliability test [84]. The six lambda measures were used to analyze the reliability and validity of the data series collected within the sample. Among the six lambda measures, lambda-2 and lambda-3 (equivalent to Cronbach’s alpha coefficients) were the most relevant reliability measures [83]. Table 6 illustrates the measurements obtained in the Guttman reliability analysis for the lambda coefficients.

Both measures, lambda-2 and lambda-3, indicate excellent data reliability, reflecting the results’ validity.

Table 7 presents the outer weights and loadings of the exogenous variables. All outer weights and loadings are significant for the chosen formative model.

According to Hair et al. [84] and Garson [83], for a formative model, an important indicator is SRMR. The SRMR of our model is 0.032 (below 0.08). Additionally, the NFI (normed fit index) has an excellent value of 0.965 (over 0.9). R-square indicates substantial effects for sustainability (0.893) and digital transformation (0.847). The formative model should not display extreme multicollinearity, according to Hair et al. [84]. Therefore, multicollinearity may be problematic if the variance inflation factor (VIF) exceeds 5. The proposed model is also relevant considering the multicollinearity criterion (Table 8).

Running a bootstrapping (0.05 significance level), we determined the path coefficients and indirect effects (Table 9). All path coefficients are above 0.3, highlighting solid causal relationships. In addition, values of *t*-statistics (above 1.6) and *p*-values (below 0.050) underline the model’s robustness [83].

Direct and indirect relationship analysis exerted by the managerial accounting roles on sustainability leads us to conclude that Hypothesis H2 is valid. Managerial accounting significantly influences the sustainable development (0.924), and digital transformation exerts a partial mediating effect (0.465). Digital transformation has increased the role in the sustainable development of managerial accountants. Table 9 also presents the significant direct effects of the change in the paradigm of managerial accounting on sustainable development, considering not only the financial aspects but also non-financial measures of health organizations. The roles of managerial accounting in sustainable organizational development decision-making are increasingly more significant due to digital transformation.

## 5. Discussion

The organization’s position towards sustainability modifies the managerial accounting roles throughout the production cycle. Currently, production cycles end with sustainability reporting that complements financial reporting. In addition, more and more organizations are integrating reporting, raising the importance of managerial accounting. Sustainability reporting improves accounting information systems for financial and non-financial reporting and the expansion of reporting measures by focusing on social and environmental vectors. Furthermore, using new technologies, organizations can achieve real-time reporting accessible to all categories of stakeholders, interactive and integrated [85].

The IFAC [29] also points out that the role of managerial accounting professionals is much more complex. Accountants not only prepare financial and sustainability reports but also have an active role in ensuring sustainability. As it results from the investigation of the H1 hypothesis, the respondents consider that the roles of creators and enablers of sustainable value are the most important, having a more significant influence on the three drivers of sustainability in the healthcare industry. As the IFAC [28,29,85,86,87,88] and Makarenko and Plastun [15] showed, managerial accounting professionals adapt to the business model where sustainability is the key. This view of the functional managerial accounting roles is compatible with managerial decision-making levels in the organization. Managerial accounting professionals are creators of sustainable values at the strategic level and enablers of sustainable values at the operational level. At the reporting level, they are preservers and reporters.

The investigation of the H2 hypothesis led to the identification of a significant influence of managerial accounting on sustainable development in the healthcare industry and a mediating effect of digital transformation. The results are in line with the findings of Willekes et al. [3], which showed that it is increasingly necessary to create an efficient accounting information system that provides data not only on financial values but also on social and environmental values [47,48,49]. Moreover, other authors believe that sustainability accounting and reporting must be based on managerial accounting tools and innovations offered by digital transformation [5,22,23,50,51], expanding the managerial accounting roles in ensuring organizational sustainability.

The testing and validation of the two hypotheses provide a clear picture of the importance of digitized managerial accounting in ensuring sustainable development, not only by assuming the roles of preservers and reporters of sustainable value but also by increasing the importance of the roles of sustainable value creators and enablers.

### 5.1. Theoretical Implications

Although there is a consensus in the academic and professional literature that managerial accounting professionals must be actively involved in sustainability accounting, uncertainty concerning the roles of accountants in sustainability is still present [43,44]. Bebbington and Larrinaga [52] (p.16) called for support to introduce “a different approach to research (in methodological terms)” to overcome “the limitations of our current accounting approaches to knowledge and its application”. Williams [45] suggested that new skills are needed specific to sustainability issues using new digital technologies to increase the level of involvement of management accountants in sustainability accounting. In addition, Schaltegger et al. [22] argued that managerial accounting professionals must integrate into their work measurement and management tools and IT solutions based on digital technologies to address sustainability issues explicitly. This paper proposes a theoretical model for analyzing the roles played by managerial accounting (proposed by the IFAC) [28] in the three pillars of sustainability based on the innovations provided by digital transformation.

### 5.2. Empirical and Managerial Implications

This paper also provides critical managerial and practical implications. It provides managerial accounting professionals and other practitioners within healthcare organizations with a clear and coherent perspective on the challenges and opportunities presented by new digital technologies in adopting the roles of creators and enablers of sustainable values. Furthermore, in this study, we emphasize the need for management accountants to assume more critical roles in developing the healthcare organization’s sustainability agenda and identify the resources and the effects on the three sustainability drivers of active involvement. These results offer evidence to managerial accounting professionals that allow them to participate in sustainability initiatives, as ensuring sustainability is a fundamental process that requires a digitized accounting information system to be carried out effectively. The roles of managerial accounting in decision-making in the healthcare industry’s sustainability field are increasingly more significant due to digital transformation.

### 5.3. Limitations and Further Research

Although the results of this exploratory study are robust, several research limitations can be addressed in future empirical investigations. First, a research limit is given by the small number of variables included. The research results illustrate the respondents’ perceptions regarding the roles of managerial accounting in creating economic value and the influences on the pillars of sustainable development. Based on the results of the exploratory research, we intend to expand the number of variables in future research by detailing the characteristics of managerial accounting roles and the features that illustrate the three pillars of sustainable development. Second, the sample selected from among Romanian managerial accountants in the healthcare industry has limited representativeness, but this study lays the groundwork for more in-depth future research. The study can be extended to managerial accountants from other fields and countries. Third, the cross-sectional nature is a limitation that a longitudinal approach in subsequent research can overcome. Future research may also consider other frameworks concerning the managerial accounting roles of professionals.

## 6. Conclusions

Integrating sustainability organizations’ strategies require reformatting the accounting information system based on new digital technologies that allow decisions grounded with sustainability drivers. Following the research undertaken, we found that new digital technologies have a beneficial influence on managerial accounting, enriching managerial accountants’ roles in the sustainable development of organizations. Although the traditional roles of reporters and preservers have remained important, managerial accounting is increasingly assuming the roles of managerial accounting. The digital transformation that has affected most fields and professions improves managerial accountants’ tools, allowing them to assume more critical roles in the organization. Through the new information provided, managerial accounting creates the opportunity for better decisions that create values for healthcare organizations. Therefore, the digital transformation of managerial accounting needs further investigation, because accounting professionals must acquire IT skills, in addition to accounting skills, to use new digital technologies integrated into complex ERP (Enterprise Resource Planning) solutions to make optimal decisions.

## Figures and Tables

**Table 1:** Questionnaire structure.

Variables	Items	Scales	References
Demographic variables	Gender	Male (1), Female (2)	
	Age	18–30 years (1), 31–45 years (2), 46–65 years (3)	
Managerial accounting roles	Creators	1 to 5 (1—non-important, 5—most important)	[15,28,29]
	Enablers		
	Preservers		
	Reporters		
Sustainability	Social	1 to 5 (1—very small, 5—very high)	
	Environment		[32]
	Financial		
Digital transformation	DTe	1 to 5 (1—minimal effect, 5—maximal effect)	[2]

Source: the authors’ design based on [2,15,28,29,32].

**Table 2:** Descriptive statistics.

	Min	Max	Mean	Std. Deviation	Skewness	Kurtosis
Gender	1	2	1.46	0.499	0.142	−1.990
Age	1	3	2.04	0.752	−0.066	−1.225
Creators	2	5	3.82	0.993	−0.388	−0.912
Enablers	1	5	3.81	0.941	−0.526	−0.388
Preservers	1	5	3.70	0.881	−0.094	−0.564
Reporters	2	5	3.72	0.975	−0.145	−1.033
Social	1	5	3.55	0.968	−0.095	−0.815
Environment	1	5	3.82	0.881	−0.484	−0.179
Financial	1	5	3.86	0.949	−0.428	−0.594
DTe	1	5	3.27	1.325	−0.187	−1.119

Source: the authors’ design using SPSS v.20.

**Table 3:** MLP model predictors.

Predictor		Predicted					
		Hidden Layer 1	Output Layer				
		H (1:1)	Social	Environment	Financial	Importance	Normalized Importance
Input Layer	(Bias)	−0.354					
	Creators	0.204				0.152	51.5%
	Enablers	0.278				0.296	100.0%
	Preservers	0.253				0.270	91.3%
	Reporters	0.363				0.282	95.6%
Hidden Layer 1	(Bias)		−1.626	−0.795	−2.333		
	H (1:1)		5.251	4.111	6.916		

Source: the authors’ design using SPSS v.20.

**Table 4:** Multivariate tests.

Effect		Value	F	Hypothesis df	Error df	Sig.	Partial Eta Squared	Noncent. Parameter	Observed Power
Intercept	Pillai’s Trace	0.121	17.932	3.000	389.000	0.000	0.121	53.795	1.000
	Wilks’ Lambda	0.879	17.932	3.000	389.000	0.000	0.121	53.795	1.000
	Hotelling’s Trace	0.138	17.932	3.000	389.000	0.000			
	Roy’s Largest Root	0.138	17.932	3.000	389.000	0.000	0.121	53.795	1.000
Creators	Pillai’s Trace	0.127	18.824	3.000	389.000	0.000	0.127	56.471	1.000
	Wilks’ Lambda	0.873	18.824	3.000	389.000	0.000	0.127	56.471	1.000
	Hotelling’s Trace	0.145	18.824	3.000	389.000	0.000	0.127	56.471	1.000
	Roy’s Largest Root	0.145	18.824	3.000	389.000	0.000	0.127	56.471	1.000
Enablers	Pillai’s Trace	0.378	78.849	3.000	389.000	0.000	0.378	236.548	1.000
	Wilks’ Lambda	0.622	78.849	3.000	389.000	0.000	0.378	236.548	1.000
	Hotelling’s Trace	0.608	78.849	3.000	389.000	0.000	0.378	236.548	1.000
	Roy’s Largest Root	0.608	78.849	3.000	389.000	0.000	0.378	236.548	1.000
Preservers	Pillai’s Trace	0.216	35.786	3.000	389.000	0.000	0.216	107.357	1.000
	Wilks’ Lambda	0.784	35.786	3.000	389.000	0.000	0.216	107.357	1.000
	Hotelling’s Trace	0.276	35.786	3.000	389.000	0.000	0.216	107.357	1.000
	Roy’s Largest Root	0.276	35.786	3.000	389.000	0.000	0.216	107.357	1.000
Reporters	Pillai’s Trace	0.376	78.050	3.000	389.000	0.000	0.376	234.150	1.000
	Wilks’ Lambda	0.624	78.050	3.000	389.000	0.000	0.376	234.150	1.000
	Hotelling’s Trace	0.602	78.050	3.000	389.000	0.000	0.376	234.150	1.000
	Roy’s Largest Root	0.602	78.050	3.000	389.000	0.000	0.376	234.150	1.000

Source: the authors’ design using SPSS v.20.

**Table 5:** Tests of between-subject effects.

Source	Dependent Variable	Type III Sum of Squares	df	Mean Square	F	Sig.	Partial Eta Squared	Noncent. Parameter	Observed Power ^d^
Corrected Model	Social	281.463 ^a^	4	70.366	310.113	0.000	0.760	1240.451	1.000
	Environment	191.170 ^b^	4	47.793	161.457	0.000	0.623	645.829	1.000
	Financial	275.565 ^c^	4	68.891	334.551	0.000	0.774	1338.205	1.000
Intercept	Social	4.690	1	4.690	20.670	0.000	0.050	20.670	0.995
	Environment	6.485	1	6.485	21.908	0.000	0.053	21.908	0.997
	Financial	0.841	1	0.841	4.084	0.044	0.010	4.084	0.522
Creators	Social	2.367	1	2.367	10.430	0.001	0.026	10.430	0.896
	Environment	12.322	1	12.322	41.627	0.000	0.096	41.627	1.000
	Financial	4.789	1	4.789	23.259	0.000	0.056	23.259	0.998
Enablers	Social	38.783	1	38.783	170.924	0.000	0.304	170.924	1.000
	Environment	0.226	1	0.226	0.763	0.383	0.002	0.763	0.140
	Financial	18.296	1	18.296	88.848	0.000	0.185	88.848	1.000
Preservers	Social	10.260	1	10.260	45.219	0.000	0.104	45.219	1.000
	Environment	7.780	1	7.780	26.283	0.000	0.063	26.283	0.999
	Financial	15.093	1	15.093	73.293	0.000	0.158	73.293	1.000
Reporters	Social	18.493	1	18.493	81.503	0.000	0.172	81.503	1.000
	Environment	35.889	1	35.889	121.243	0.000	0.237	121.243	1.000
	Financial	24.559	1	24.559	119.264	0.000	0.234	119.264	1.000

(^a^) R Squared = 0.760 (Adjusted R Squared = 0.758). (^b^) R Squared = 0.623 (Adjusted R Squared = 0.619). (^c^) R Squared = 0.774 (Adjusted R Squared = 0.772). (^d^) Computed using alpha = 0.05. Source: the authors’ design using SPSS v.20.

**Table 6:** Guttman’s reliability analysis.

Lambda	Values
1	0.824
2	0.947
3	0.942
4	0.947
5	0.938
6	0.953

Source: the authors’ design using SPSS v.20.

**Table 7:** Outer weights.

	Digital Transformation		Managerial Accounting		Sustainability	
	Outer Weights	Outer Loadings	Outer Weights	Outer Loadings	Outer Weights	Outer Loadings
Creators			0.167	0.784		
Enablers			0.377	0.833		
Preservers			0.281	0.716		
Reporters			0.409	0.865		
Financial					0.499	0.954
Environment					0.102	0.708
Social					0.472	0.942
Dte	1.000	1.000				

Source: the authors’ design using SmartPLS v3.0.

**Table 8:** Multicollinearity of endogenous variables.

	VIF
Creators	2.080
Enablers	1.834
Preservers	1.393
Reporters	1.968
Financial	3.558
Environment	2.224
Social	2.947
Dte	1.000

Source: the authors’ design using SmartPLS v3.0.

**Table 9:** Path coefficients.

Effects	Path	Original Sample	Sample Mean	Standard Deviation	*t* Statistics	*p* Values
Direct	Managerial accounting → Digital transformation	0.921	0.921	0.008	117.639	0.000
	Managerial accounting → Sustainability	0.459	0.463	0.050	9.127	0.000
	Digital transformation → Sustainability	0.506	0.502	0.050	10.178	0.000
Specific	Managerial accounting → Digital transformation → Sustainability	0.465	0.462	0.046	10.040	0.000
Total	Managerial accounting → Digital transformation	0.921	0.921	0.008	114.906	0.000
	Managerial accounting → Sustainability	0.924	0.925	0.008	122.049	0.000
	Digital transformation → Sustainability	0.506	0.501	0.052	9.810	0.000

Source: the authors’ design using SmartPLS v3.0.

## Data Availability

Not applicable.

## Appendix A

**Table A1:** Questionnaire items.

Variables	Items
Demographic variables	What is your gender?
	What is your age range?
Managerial accounting	On a scale from 1 to 5 (1—non-important, 5—most important), what do you think is the importance of the value creator role of managerial accounting?
	On a scale from 1 to 5 (1—non-important, 5—most important), what do you think is the importance of the value enabler role of managerial accounting?
	On a scale from 1 to 5 (1—non-important, 5—most important), what do you think is the importance of the value preserver role of managerial accounting?
	On a scale from 1 to 5 (1—non-important, 5—most important), what do you think is the importance of the value reporter role of managerial accounting?
Sustainable development	On a scale of 1 to 5 (1— very small, 5— very high), what do you think is the contribution of the economic pillar to sustainable organizational development?
	On a scale of 1 to 5 1— very small, 5— very high), what do you think is the contribution of the social pillar to sustainable organizational development?
	On a scale of 1 to 5 1— very small, 5— very high), what do you think is the contribution of the environmental pillar to sustainable organizational development?
Digital transformation	On a scale from 1 to 5 (1—minimal extent, 5—maximal extent), what is the degree of use of the new digital technologies in managerial accounting activities?

Source: own construction based on [2,15,28,29,32].
